# Exploring Fungal Biodegradation Pathways of 2,4-D: Enzymatic Mechanisms, Synergistic Actions, and Environmental Applications

**DOI:** 10.1021/acsomega.5c05161

**Published:** 2025-08-25

**Authors:** Ana Caroline Barros do Nascimento, Nildo A. Nhampossa, Thaís P. Félix, Ivaldo Itabaiana, Rodrigo P. do Nascimento

**Affiliations:** † Department of Biochemical Engineering, School of Chemistry, Federal University of Rio de Janeiro (UFRJ), 21941-909 Rio de Janeiro, RJ, Brazil; ‡ Center for Health Sciences, Federal University of Rio de Janeiro (UFRJ), Av. Carlos Chagas Filho, 373, 21941-590 Rio de Janeiro, RJ, Brazil

## Abstract

2,4-D (2,4-dichlorophenoxyacetic acid) is one of the most widely used herbicides globally, effectively controlling broadleaf weeds in various agricultural systems. However, its persistence in the environment and potential health risks raise significant concerns, demanding efficient and sustainable detoxification strategies. This review critically examines the fungal biodegradation of 2,4-D, with a specific focus on the enzymatic pathways mediated by laccases, manganese peroxidases, and lignin peroxidases—key oxidative enzymes involved in the transformation of chlorinated aromatic compounds. Laccases initiate degradation by oxidizing phenolic structures and generating phenoxy radicals, while peroxidases contribute through the generation of reactive oxygen species that facilitate the cleavage of stable C–Cl and C–C bonds. The synergistic activity of these enzymes enhances degradation efficiency and expands the range of metabolizable compounds. Additionally, we explore the influence of environmental factors—such as pH, temperature, and nutrient availability—on enzymatic activity and stability. The review also discusses potential applications of intermediate metabolites, including their valorization in pharmaceutical and agrochemical industries. By integrating recent experimental findings and mechanistic insights, this work provides a comprehensive overview of fungal enzymatic systems for 2,4-D degradation and highlights their potential in advancing bioremediation strategies.

## Graphical abstract

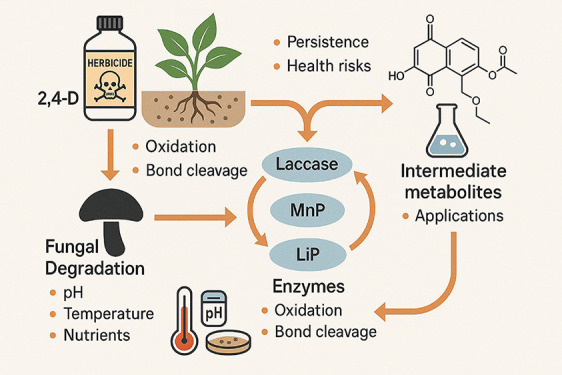

## 1 Introduction

The increase in global life expectancy has directly influenced industrialization to meet rising energy and food demands. In this context, the use of pesticides has been pivotal in modern agriculture, where pest control is a crucial strategy for enhancing crop productivity.^1^

Pesticides are substances or mixtures designed to prevent, destroy, repel, or mitigate pests.^2,3^ Although these chemicals effectively target pest organisms, they also pose risks to nontarget organisms and can cause various health issues in humans.^4^ The three primary categories of pesticides — herbicides, fungicides, and insecticides — account for approximately 47% of global pesticide usage.^5^ These pesticides effectively reach less than 10% of their intended targets.^6^ Due to their high environmental persistence, these chemicals have significant potential for bioaccumulation and biomagnification in human and animal tissues. They are toxic as they interfere with humans’ and wildlife’s endocrine and reproductive systems.^7–9^

The herbicide 2,4-D, whose molecular structure is depicted in Figure 1, is readily absorbed by plant leaves and can penetrate lipid-rich cell membranes due to its lipophilic nature. This lipophilicity affects soil adsorption to organic particles, influencing its mobility and availability for microbial degradation.^10^ Lipophilic compounds tend to accumulate in the fatty tissues of living organisms. While 2,4-D is not highly bioaccumulative, its lipophilicity can impact the retention of its metabolites in both aquatic and terrestrial organisms. This characteristic can increase the herbicide’s toxicity to marine life by allowing it to penetrate cell membranes and cause biological damage. In humans, 2,4-D’s lipophilicity can enhance absorption through the skin and mucous membranes, thereby elevating the risks associated with occupational and environmental exposure.

**Figure 1:**
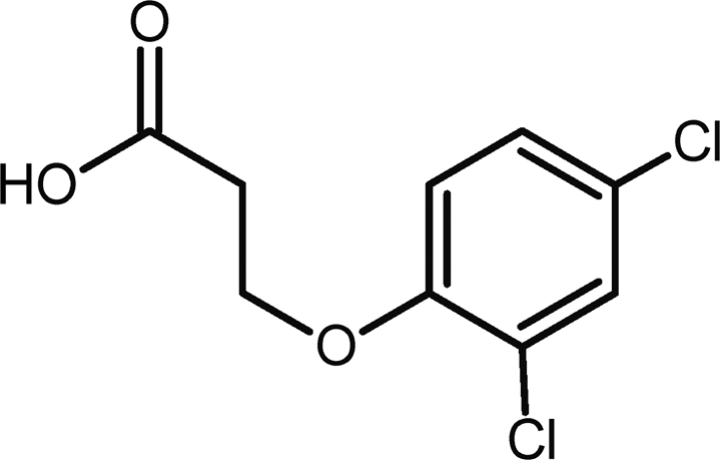
Chemical structure of 2,4-D.

Brazil is the largest consumer of pesticides globally, with herbicides being the most widely used class. Among these, 2,4-dichlorophenoxyacetic acid (2,4-D) is the second most applied herbicide. It is used extensively across various crops, including rice, sugar cane, coffee, corn, soybean, and wheat. As a member of the organochlorine and phenoxy families, 2,4-D is classified as highly toxic by the World Health Organization (WHO)^11^ examined methanogenic enrichment cultures from Amazonian topsoil and deep soil, demonstrating the biotransformation of 2,4-D into 4-chlorophenol and phenol. Despite its widespread use, 2,4-D is the third most applied pesticide in Brazilian soils, primarily used for controlling broadleaf weeds in agricultural plantations and pastures.^12^ The herbicide’s high mobility and long half-life under anoxic conditions pose a significant risk of groundwater contamination.^13^ Therefore, bioremediation, mainly through fungal and bacterial processes, is a promising approach to address anoxic contamination caused by 2,4-D. However, further research is required to enhance the understanding of anaerobic biodegradation pathways for this herbicide.

Developed in the 1940s, 2,4-D quickly became popular in agriculture due to its effectiveness in selectively targeting broadleaf weeds while leaving most grasses unharmed. The action of 2,4-D involves inducing the expression of auxin-responsive genes, which leads to the biosynthesis of ethylene and abscisic acid (ABA) hormones. Both ethylene and ABA inhibit cell division, stimulate leaf senescence, and ultimately result in growth inhibition, tissue damage, and plant death.^14,15^ Over the years, its application has increased significantly due to the need for an alternative herbicide against glyphosate-resistant weeds. Its use has expanded beyond agriculture to residential and industrial settings.^10,16–18^

The significant accumulation of 2,4-D in the soil and the food consumed carries this herbicide into the human body and animals.^9^ Due to this, potential toxic effects on the endocrine system in humans have been reported in the literature.^8^ Other studies have shown various effects on animals, such as fetal anomalies and damage to the kidneys, thyroid, adrenal glands, eyes, and ovaries. In addition to its toxicity to humans and animals, 2,4-D in the environment can be transported through various mechanisms, such as rainwater, volatilization, crop removal, leaching, plant uptake, chemical degradation, adsorption, runoff, microbial degradation, and photodecomposition processes.^19,20^

The persistence of 2,4-D depends on environmental conditions. It can be broken down by microbial activity,^21^ often leading to less toxic degradation products. However, more harmful compounds may be produced. Microbial metabolism plays a key role in the transformation or degradation of pesticides.^2,22^ Nevertheless, low pH, temperature, moisture, and soil characteristics extend its persistence and slow its degradation.^11,23,24^ Concerns about environmental degradation and the adverse effects of the use of 2,4-D have led to the search for efficient remediation strategies. In this context, biodegradation has been a promising alternative. Among the microorganisms present in contaminated soils, filamentous fungi have been reported to have the ability to degrade toxic compounds, including 2,4-D.^18^ Their remarkable ability to adapt to different environments, their ability to secrete various enzymes, and their high mutagenicity make filamentous fungi very attractive for these purposes.

Biodegradation by filamentous fungi involves the action of specific extracellular enzymes that can cleave the herbicide in coordination or isolation, generating various intermediate compounds that may be less toxic. These processes can convert the herbicide into less harmful products or completely mineralize it.^23,25^ Some fungi capable of degrading herbicides can thrive in high concentrations of these contaminants. They utilize the intermediates produced, along with existing soil nutrients, as a source of energy for their growth by incorporating them into their metabolism.^19,26^

The degradation of 2,4-D by filamentous fungi has been the focus of scientific research aimed at understanding the mechanisms involved and exploring the potential of these microorganisms for remediating contaminated soil and water. Key aspects of this research include identifying and selecting filamentous fungi capable of degrading 2,4-D, studying their characteristics and potential genetic improvements, optimizing cultivation conditions, and evaluating degradation products.^26–28^

Although significant progress has been made in studying the biodegradation of 2,4-D by filamentous fungi, several challenges remain. Understanding the factors influencing degradation efficiency is crucial for developing effective remediation strategies. Additionally, scalability and economic feasibility are essential considerations.^24,29–31^

This critical review evaluates and maps recent advances in the biodegradation of 2,4-D by filamentous fungi, addressing the mechanisms involved, experimental studies conducted, factors affecting degradation efficiency, and prospects in this field. Furthermore, it proposes practical applications for the metabolites formed to highlight their potential for valorization. Through this analysis, we aim to advance knowledge and develop sustainable solutions for remediating herbicide contamination.

## 2 Contamination Environmental Impacts of 2,4-D

Due to the continuous growth and dynamic nature of living systems compared to relatively static soil systems, chemical residues are generally degraded or diluted more rapidly in living organisms. Consequently, pesticides persist in the soil longer than in plants or animals. The duration of pesticide presence in soil is influenced by various environmental factors and specific characteristics of the pesticide itself.^32^ These factors significantly affect the persistence of pesticides in soil and can be categorized as follows:^33^ (i) Physical, Chemical, and Biological Characteristics of the Soil–these include the content of available organic matter, the diversity of microorganisms present at the site, pH, moisture, and temperature; (ii) Characteristics of the Contaminant–these include volatility, water solubility, and the concentration used; (iii) Pesticide Application Method: The mode of application on the plantation also plays a role.^3,4,22,34^

Figure 2 illustrates the potential routes of 2,4-D contamination in the environment, from its application to the possible exposure of humans and animals.

**Figure 2:**
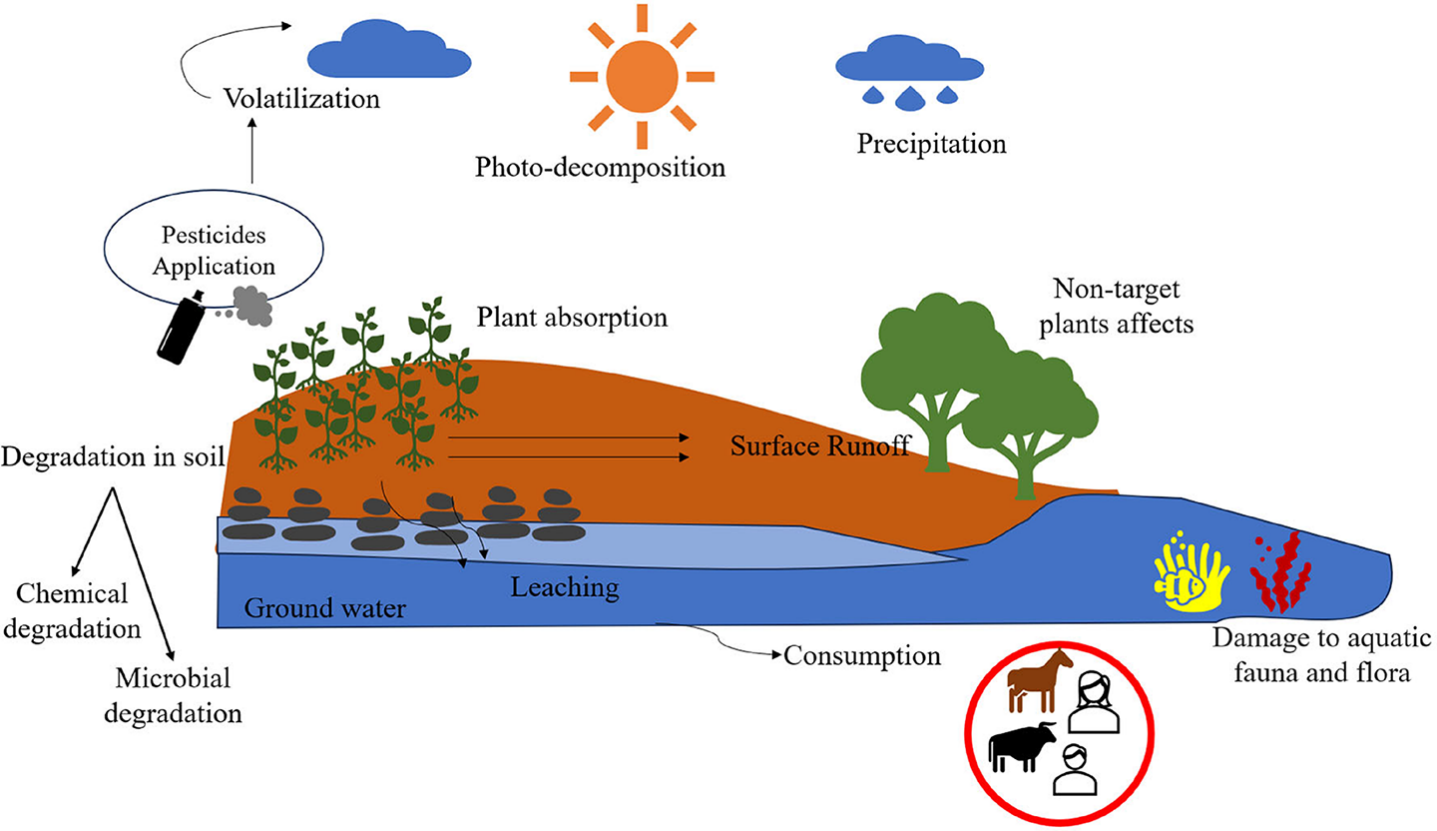
Cycle of 2,4-D in the environment following its application (Figure created by the authors).

Contamination of soil and water resources by 2,4-D can occur through several pathways. The primary route of contamination is the direct application of the herbicide to the soil surface or its dispersion via spray drift during application.^10,19^ Additionally, leaching can occur when 2,4-D percolates downward through the soil profile, potentially reaching groundwater sources. Surface runoff from treated areas can also transport the herbicide into nearby water bodies, contaminating water.^35,36^

The process of soil and water contamination involves several stages, with the three primary ones being immediate contamination following herbicide application, leaching through the soil profile, and surface runoff induced by rainwater.^2,22^

In the primary route, the herbicide is applied to the soil surface through spraying, and the physicochemical characteristics of the environment influence its mobility within the soil. The absorption of 2,4-D is affected by several factors. Generally, soils with higher organic matter content have a greater capacity to adsorb 2,4-D, which reduces the herbicide’s penetration into deeper soil layers. Conversely, the herbicide exhibits increased mobility in sandy soils with low organic matter content and a higher potential for leaching.^37,38^

Once in the soil, 2,4-D can undergo biodegradation, primarily through the activity of soil microorganisms. Microbial activity is crucial in breaking down the herbicide into metabolites, which can vary in toxicity and persistence depending on the specific microbial populations present.^24^ The microbial decomposition of 2,4-D in the soil aims to detoxify the molecule by increasing its polarity and water solubility, thereby facilitating its breakdown and assimilation. This process can involve several reactions, including hydroxylation, cleavage of the acid’s side chain, decarboxylation, and ring-opening.^14,22^

The second stage involves the compound percolating through different soil horizons, potentially reaching groundwater resources.^34^ This process can extend the compound’s reach, potentially affecting organisms beyond weeds, including nontarget plants and insects. During the leaching stage, key factors influencing 2,4-D movement include the herbicide concentration, oxygen availability, and soil pH.^19^ In soils with a pH ranging from 5 to 8, which encompasses most soil types, 2,4-D primarily exists as an anion, resulting in minimal interaction with the soil. As the pH increases, this ionic bond weakens, leading to more significant leaching of the compound.^37,39,40^

Finally, rainfall-runoff can transport the compound across the soil surface to nearby water bodies. Once in the water, the half-life of 2,4-D is estimated to be about 15 days under aerobic conditions but can range from 41 to 333 days under anaerobic conditions. This extended persistence in the environment can lead to further contamination of water bodies, posing potential risks to humans and animals who may come into contact with or consume the herbicide.^13,38^ Table 1 presents the tested lethal doses of 2,4-D and the signs of toxicity observed in animals and humans that may be associated with chronic exposure to the herbicide, as reported by the National Pesticide Information Center in the United States.

**Table 1:** Data on the Lethal Dose for 2,4-D in Various Animal Species Conducted in the Laboratory and an Analysis of Toxicity Signs for Different Levels of Herbicide Exposure^a^

toxicity	organism	DL_50_	signs of toxicity
oral	rat	138 mg/kg	death
	mouse	693–1646 mg/kg	
inhalation	mouse	0.78 mg/L	
dermal	rabbit	1829 mg/kg	
fetal abnormalities	rat	90 mg·day/kg	
chronic exposure	domestic dog	ND	potential association between animals’ exposure to 2,4-D, when used by their owners in gardens, and the development of lymphoma in these animals
	human	ND	adverse impacts on the endocrine system and neuromuscular functions can persist for extended periods, ranging from weeks to months, with some cases resulting in permanent effects
immediate exposure	domestic dog	ND	myotonia, vomiting, weakness, inappetence, anorexia, ataxia, salivation, diarrhea, lethargy, and convulsions
	human	ND	**Oral exposure:** symptoms may include vomiting, diarrhea, headache, confusion, and aggressive or bizarre behavior. A peculiar odor may sometimes be noted on the breath. Additionally, skeletal muscle injury and renal failure can occur. **Dermal exposure:** potential effects may include irritation. Inhalation exposure could result in symptoms such as coughing and a burning sensation in the upper respiratory tract and chest

a Adapted from refs 3, 8, and 41.

Although the carcinogenicity of 2,4-D has not been definitively proven, there is ongoing concern about a potential association between prolonged exposure to the herbicide and the development of cancers, such as lymphomas, in workers. Researchers have not established a clear link between 2,4-D and cancer in humans. This challenge is further complicated because 2,4-D is frequently used in combination with other herbicides, making it difficult to isolate its specific effects.^3,8^ Some studies have suggested a potential association between non-Hodgkin lymphoma and exposure to 2,4-D alone, while other research has not confirmed this link. Logging, an ordinary human activity in forested areas, has led to extensive studies on environmental degradation and its impacts on wildlife. Reduced impact logging practices, especially those adhering to certification standards, may help balance forest production with biodiversity conservation. Additionally, occupational exposure to 2,4-D has been linked to reductions in sperm quantity and viability, with malformations persisting even after exposure has ceased. While the mutagenic potential of 2,4-D is not fully understood, DNA damage has been observed in hamsters, and alterations in gene expression have been noted.^9,42^ In the Figure 3, the main effects of exposure to the herbicide 2,4-D are presented based on studies conducted by refs 8,9,41–43 .

**Figure 3:**
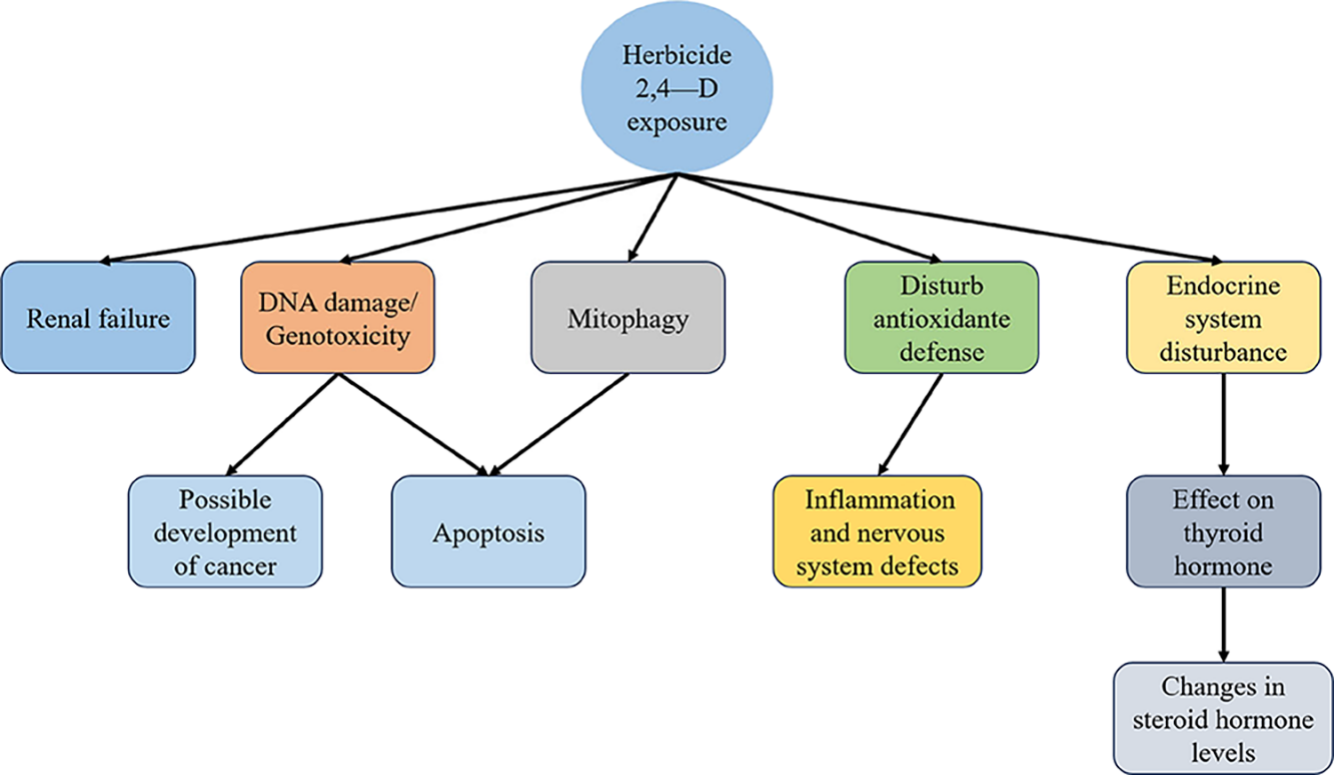
Effects of herbicide 2,4-D exposure in diverse organisms.

Numerous cases of 2,4-D detection in water bodies from rural and urban areas have been reported in the literature. In the United States, a survey by the EPA found that 49.3% of drinking water samples contained 2,4-D at concentrations up to 2 ppb. Although this concentration is below the accepted regulatory limit of 70 ppb, nearly half of the samples tested positive, underscoring the potential risk associated with water source contamination.^3,8^ According to the publication, soil washing is an alternative method for removing 2,4-D, and water and surfactants are employed to address the herbicide’s lipophilic nature. However, this technique primarily transfers the contaminant rather than eliminating it. To achieve complete remediation, additional treatments, such as Fenton and photo-Fenton processes (Advanced Oxidation Processes, AOPs), are necessary^44^ for ozonation, activated carbon adsorption,^45^ and electrochemical techniques.^46–49^ All these processes involve contaminant transfer, which can become impractical for large contaminated areas. In such cases, biodegradation emerges as the most effective technique for in situ treatment, providing a more sustainable solution for managing extensive contamination.

## 3 Biodegradation

Biodegradation leverages biological agents—such as bacteria, fungi, plants, and enzymes—to transform, reduce, or mineralize environmental contaminants. This process utilizes the natural metabolic activities of these organisms to break down pollutants into less harmful substances.^17,26,27^ This method is a sustainable alternative for addressing soil and water contamination, offering several advantages over physical and chemical remediation methods. Biodegradation generally requires less energy and chemical input, minimizes the risk of secondary pollution, and supports natural recovery processes, making it a more environmentally friendly and cost-effective solution:^6,54,55^

- Living organisms and their enzymes are employed to degrade contaminants, whereas chemical remediation methods often require more chemical additives and have lower selectivity. Enzymes, in contrast, are known for their high selectivity in targeting specific pollutants.
- Biological systems typically result in fewer adverse environmental impacts compared to chemical and physical methods, which can produce more toxic residues or cause additional harm to the ecosystem.
- Moreover, biodegradation can be more efficient in natural or remote areas where implementing chemical and physical methods may be challenging and costly.

The bacterial degradation pathways of the herbicide 2,4-D are well-defined and extensively documented in the literature. Bacterial species that produce enzymes such as 2,4-dichlorophenoxyacetate monooxygenase (TfdA) and 2,4-dichlorophenol hydroxylase (TfdB) are essential for catalyzing the initial steps of 2,4-D decomposition. These enzymes convert the herbicide into less toxic and more hydrophilic metabolites. Bacterial genera such as Pseudomonas, Alcaligenes, and Acinetobacter are particularly noted for their efficiency in degrading 2,4-D.^24,56^

In recent years, research has focused on investigating the fungal pathways of 2,4-D biodegradation compared to bacterial pathways.^1,57,58^ Several factors contribute to this shift in emphasis. First, filamentous fungi have a more complex metabolism, enabling them to utilize a broader range of metabolic pathways. They produce various extracellular enzymes that aid in the degradation of 2,4-D. Enzymes such as peroxidases can break the chemical bonds of 2,4-D, reducing the toxicity of the resulting products to the microorganisms in the contaminated environment.^1^ Fungi are also recognized for their ability to colonize and adapt to diverse environmental conditions, making them versatile degraders. Their adaptability enables them to tolerate a broader range of pollutant concentrations, enhancing their effectiveness in Campo bioremediation efforts.^59,60^

Second, the fungal degradation process can involve multiple stages and often employs various fungal species working synergistically to degrade the herbicide. While the presence of diverse species complicates the task of determining the individual contribution of each one, their collective action enhances the efficiency of contaminant mineralization, potentially converting the herbicide into CO_2_. This cooperative behavior broadens the range of contaminants that can be degraded and underscores fungi’s value as research subjects for developing effective bioremediation strategies.^58,60,61^

The long-term ecological impacts of introducing specific fungal species or their enzymes into contaminated environments for 2,4-D bioremediation are complex and must be carefully considered. While fungal-based remediation offers an eco-friendly alternative to chemical treatments, introducing non-native or genetically modified strains may disrupt native microbial communities and ecological balances. Studies have shown that fungal activity can alter soil microbiota, nutrient cycling, and organic matter decomposition processes, potentially reducing biodiversity or favoring opportunistic organisms over native species.^1,2^ Furthermore, residual enzyme activity or metabolic byproducts—such as chlorinated phenols or quinones—may persist in the environment and impact nontarget organisms over time. To mitigate such risks, researchers recommend using well-characterized native fungal strains, applying immobilized enzymes to limit environmental dispersion, and performing site-specific ecological assessments before field application.^1,3,4^ Furthermore, residual enzyme activity or metabolic byproducts—such as chlorinated phenols or quinones—may persist in the environment and impact nontarget organisms over time.^5–7^

### 3.1 Filamentous Fungi as Biodegradation Agents

The benefits of fungal-mediated biodegradation extend beyond mere pollutant removal. By reducing the persistence of 2,4-D in the environment, fungal activity helps prevent its accumulation in soil and water ecosystems, thereby protecting biodiversity and maintaining ecosystem integrity. Additionally, the degradation products formed during fungal metabolism are generally less toxic and more readily assimilated by other organisms, minimizing adverse effects on ecosystem health. Furthermore, mycoremediation offers a cost-effective and environmentally friendly alternative to conventional remediation methods that rely on harsh chemicals.^57,62–64^

The primary biochemical reactions involved in the fungal degradation of pesticides include oxidation, reduction, alkylation, dealkylation, dehalogenation, dehydrogenation, hydroxylation, amide and ester hydrolysis, ether cleavage, ring cleavage, condensation, and conjugate formation. These diverse reactions facilitate the breakdown of pesticide compounds, aiding their detoxification and removal from the environment.^23,24,61,65^ It is important to note that some fungi can degrade specific compounds but may not be able to degrade their intermediate metabolites further, or vice versa. This indicates that different enzymes are involved in these processes, reflecting the genetic capacities of each fungal species to express specific enzymes in response to the presence of 2,4-D.^21,66^ Table 2 summarizes studies on the degradation of 2,4-D using filamentous fungi and the various concentrations applied during these analyses. These studies reveal that the principal metabolite formed, 2,4-dichlorophenol (2,4-DCP), has a significantly lower degradation capacity than the original compound. This metabolite is more toxic than 2,4-D, contributing to its increased persistence and difficulty in degradation. Additionally, research evaluating the degradation efficiency of 2,4-DCP itself has mainly yielded unsatisfactory results, highlighting the challenges posed by the metabolite’s high toxicity and low biodegradability. Table 3 lists fungal species documented in the literature for their ability to degrade 2,4-D.

**Table 2:** Methods for Remediating Environments Contaminated with 2,4-D, Advantages and Disadvantages

remediation method	techniques	advantages	disadvantages	ref
chemical	oxidation	effective treatment of high concentration	generate harmful products or require additional treatment steps to ensure complete degradation	^47^
	adsorption	effective in adsorbing the contaminants from soil or water	the adsorbents may become saturated over time and require regeneration or disposal	^40^
	hydrolysis	effective method	requires the use of strong bases or acids	^50^
physical	soil washing	a practical method of removing 2,4-D from soil	requires significant volumes of water and generates wastewater that needs proper treatment	^51^
biological	biodegradation	environmentally friendly and sustainable for the long-term	it takes longer than chemical and physical methods, and the success depends on site-specific factors	^27^
	phytoremediation	reducing the concentration of soil and water	the effectiveness of degradation may vary, and there is a risk of incomplete degradation	^52^
	bioaugmentation	accelerate the 2,4-D degradation and improve remediation	it takes longer than chemical and physical methods, and the success depends on site-specific factors	^53^
	bioaccumulation	it can use the biomass of living and dead microorganisms	it takes longer than chemical and physical methods, and the success depends on site-specific factors	^24^

**Table 3:** Fungi Responsible for 2,4-D Degradation^a^

fungi	degradation level	2,4-D concentration	ref
*Trichoderma viride*	++	600 ppm	^26^
*Trichoderma koningi*	++		
*Penicillium chrysogenum*	+++		
*Lentinus crinitus*	+	22 ppm	^24^
*Penicillium crustosum*	+++	ND	^67^
*Aspergillus niger*	+++	ND	^68^
*Phanerochaete chrysosporium*	++++	40 ppm	^69^
*Aspergillus penicillioides*	+++	ND	^21^
*Acremonium murorum*	+		
*Mortirella isabelina*	+		
*Cladobotryum verticilatum*	+		
*Fusarium moniliforme*	+		
*Rhizoctonia solani*	–+	ND	^66^
*Trichoderma harzianum*	–+	2,5 ppm	^70^
*Mortierelle. genevensis*	+++		^21^
*Chrysosporium pannorum*			
*Phoma glomerata*			
*Cladosporium cladosporiodes*	++		
*Drechslera spicifera*			
*Penicillium atramentosum*			
*Cunnighamella ellegans*			
*Mortierella isabelline*			
*Syncephalastrum racemosum*			

a –+ < 25%; + € [25%;49%]; ++ € [50%;74]; +++ € [75%;89%]; ++++ >90% - ND: no data - w/w.

Species such as *Phanerochaete chrysosporium*, *Penicillium crustosum*, *Aspergillus niger*, and *Aspergillus penicillioides* demonstrated high efficiency in degrading 2,4-D, even at elevated concentrations or in studies where the concentration was not specified. On the other hand, fungi such as *Phoma glomerata*, *Drechslera spicifera*, and *Cunninghamella elegans* showed no significant activity against the herbicide. These findings highlight the importance of selecting specific strains with high degradative potential for application in bioremediation processes of pesticide-contaminated environments.

Filamentous fungi, renowned for their diverse metabolic capabilities, thrive in various environments, including those contaminated with 2,4-D.^1,60^ Some species have evolved to utilize this xenobiotic as a carbon and energy source, aiding in its degradation. Although soil fungi show promising potential for degrading herbicides and their intermediates,^24,26^ mechanistic insights into the fungal degradation of 2,4-D are still relatively incipient.^2,17^

The availability of nutrients and the pH of the environment are critical factors influencing the enzymatic degradation of 2,4-D by fungi. Several fungal species exhibit enhanced degradation performance when supplemented with cosubstrates such as sucrose, glucose, or organic acids, which serve as additional carbon and energy sources to support metabolic activity. For instance, *Penicillium chrysogenum* demonstrated the ability to tolerate and degrade higher concentrations of 2,4-D in the presence of sucrose compared to other carbon sources.^8^ Additionally, the pH significantly affects enzyme stability and activity, with optimal degradation typically occurring under near-neutral conditions. Deviations from this range can lead to reduced enzyme efficiency or denaturation, thereby limiting the overall bioremediation potential.^9^

Central to the biodegradation of 2,4-D by fungi are enzymatic processes catalyzed by dioxygenases, dehalogenases, and hydrolases. Dioxygenases initiate the degradation by introducing oxygen into the aromatic ring of 2,4-D, facilitating subsequent reactions that lead to mineralization. Given the diverse metabolic capabilities of filamentous fungi, they emerge as prime candidates for applications in pollutant degradation.^26^ Mycoremediation—a specialized subset of bioremediation utilizing fungi—has recently gained recognition as a promising approach for mitigating 2,4-D contamination. Fungi such as white rot fungi, including *Phanerochaete chrysosporium*, and certain *Trametes* and *Pleurotus* species have demonstrated remarkable efficiency in degrading 2,4-D and its derivatives. When introduced into contaminated soil or water environments, these fungi thrive and effectively break down 2,4-D, reducing environmental impact. For example, *Aspergillus niger* has been shown to degrade 2,4-D in studies by Faulkner and Woodcock,^68,71,72^ who investigated various microbial strains and soil suspensions, highlighting the importance of fungal species in soils. Fournier and Catroux^73^ also suggested that analyzing the degradation of pesticides in the presence of an additional carbon source could be crucial for characterizing the biodegradability of substances across different strains. This approach helps better understand how varying conditions influence the effectiveness of biodegradation processes.

Vroumsia et al.^66^ conducted tests to assess the ability of 90 fungal strains to degrade 2,4-D and 2,4-DCP. After 4 days of cultivation in a synthetic liquid medium, *Aspergillus penicillioides* and *Umbelopsis isabellina* (formerly *Mortierella isabellina*) were identified as the most effective species for degrading 2,4-D, while *Chrysosporium pannorum* and *Mucor generensis* proved to be the most efficient at degrading 2,4-DCP. The authors concluded that the degradation response of the strains varied by taxonomic group, with 2,4-DCP being more accessible to fungal degradation than 2,4-D. They suggested that the lower accessibility of 2,4-D for fungal degradation compared to 2,4-DCP could be attributed to its ether linkage and a nonfree phenolic hydroxyl group. On the other hand, *Penicillium* species isolated from soil contaminated with 2,4-D demonstrated significant potential for degrading this herbicide. According to Joshi et al.,^74^ it was observed that 2,4-D is toxic to soil mycobiota, and only *Aspergillus* sp. utilizes 2,4-D as a carbon source.

Ferreira-Guedes et al. reported that a strain of *Penicillium chrysogenum* isolated from a salt mine was capable of degrading 2,4-D with high efficiency, particularly when sucrose was used as an additional carbon source, in combination with α-ketoglutarate and ascorbic acid as cosubstrates.^75^ In this study, it was found that two strains of *Penicillium crustosum* were highly effective in degrading 2,4-D in synthetic agricultural wastewater. However, the authors noted that further research is needed to explore the ability of these fungi to degrade 2,4-D in natural agricultural wastewater, taking into account specific physicochemical factors.^17^

### 3.2 Metabolic Pathways

2,4-Dichlorophenol (2,4-DCP) is the primary metabolite produced during the degradation of 2,4-D.^13,21,39,66,76–78^ It often serves as an intermediate in other metabolic pathways. However, the mechanisms and the complete set of enzymes involved at each stage of these pathways, as expressed by microorganisms, are still poorly described in the literature.

Many studies primarily focus on quantifying the degradation of 2,4-D and, in some cases, 2,4-DCP without identifying the metabolites derived from these compounds. In such cases, the main objective is identifying fungi capable of degrading these compounds, often without detailing the specific pathways involved.^13,21^ It is generally suggested that different enzymes mediate the degradation of 2,4-D and 2,4-DCP, as some fungal species can degrade 2,4-D but not 2,4-DCP, and vice versa.^21^

Compared with degradation via bacterial pathways,^14^ literature has limited information regarding the mechanisms, routes, and enzymes involved in the degradation of 2,4-D by fungi. One possible reason for this gap may be the greater diversity of enzymes secreted by these microorganisms, which leads to a broader range of metabolic pathways.^24^

The initial studies on the degradation of 2,4-D by fungi were conducted by Faulkner and Woodcock.^68,71^ These studies observed hydroxylation of the aromatic ring by *Aspergillus niger*, resulting in the formation of 2,4-dichloro-5-phenoxyacetic acid and 2,5-dichloro-4-phenoxyacetic acid. However, the specific enzymes involved in this process were not identified. Subsequent research^78^ identified two metabolites resulting from 2,4-D degradation by *Aspergillus niger*: 2,4-dichlorophenol (2,4-DCP) and 3,5-dichloro catechol (3,5-DCC). Additionally, 2,4-D was utilized as a carbon and energy source, eliminating the need for glucose supplementation in the medium. In another study, it was^79^ observed that the degradation of 2,4-DCP and 3,4-dichlorophenol (3,4-DCP) by *Penicillium frequentans* in the presence of phenol as a cosubstrate. The degradation of both compounds followed two stages: oxidation of the halogenated ring and methylation. In this study, two enzymes were detected—phenol hydroxylase (EC 1.14.1.3.7) and catechol 1,2-dioxygenase (EC 1.13.11.1)—but the specific mechanisms of these enzymes were not identified.^39^ Similar steps were observed using the species *Mortierella* sp., with the identification of a possible second pathway involving two other metabolites formed from the dehalogenation of 2,4-DCP: chlorohydroquinone and hydroquinone. Dehalogenation was also identified^77^ on the degradation of 2,4,6-trichlorophenol (2,4,6-TCP) by *Phanerochaete chrysosporium*. Figure 4 presents the pathways described in the literature, the enzymes involved, and the possible metabolites resulting from degradation, as discussed in various studies.

**Figure 4:**
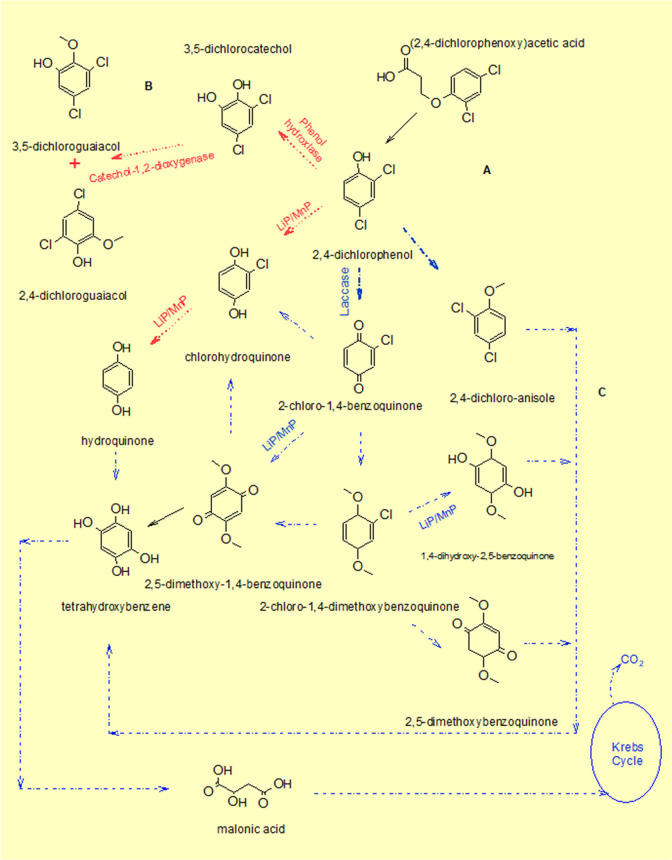
Metabolites resulting from 2,4-D degradation and possible enzymes involved in the process. Each step was organized according to the data of several sources: Step A: refs 36, 39, 46, and 75–77 . Step B: refs 39 and 77. Step C: ref 76.

Based on current evidence, the degradation of 2,4-D by fungal oxidative enzymes generally proceeds through a pathway in which laccase initially oxidizes the 2,4-D molecule, generating phenoxy radicals at the chlorinated positions of the aromatic ring.^10–12^ Subsequently, peroxidases such as manganese peroxidase (MnP) or lignin peroxidase (LiP) contribute reactive oxygen species (ROS) or high-valent iron intermediates that destabilize the electron density surrounding the carbon–chlorine bonds.^13–15^ Dechlorination then occurs either directly through ROS attack or indirectly via rearrangements and hydrolysis of the activated intermediates. Finally, further oxidation of the resulting metabolites, including chlorocatechols and quinones, leads to complete mineralization or the formation of value-added transformation products.^16^

To maximize the production of specific high-value intermediates in fungal biotransformation, it is essential to select or engineer fungal strains with enhanced enzyme expression favoring the desired compounds. Optimizing culture conditions—such as pH, temperature, nutrients, and oxygen levels—can further direct metabolic pathways toward target intermediates.^17^ The use of enzyme inducers and redox mediators helps boost catalytic activity and expand substrate range. Additionally, genetic and metabolic engineering can be applied to block undesired degradation steps, promoting accumulation of valuable metabolites. Process strategies like controlled feeding, bioreactor design, and immobilization improve stability and selectivity, while real-time monitoring enables dynamic adjustments to optimize yields. Together, these approaches enhance the efficiency and scalability of producing high-value fungal biotransformation products^9,18^

### 3.3 Extracellular Enzymes Reported on the Metabolism of 2,4-D

Fungi possess a diverse array of enzymes capable of metabolizing 2,4-D. Key enzymes involved in this process include dioxygenases, dehalogenases, hydrolases, and oxidases. Each enzyme class is crucial in breaking down 2,4-D into more straightforward, less harmful compounds. Dioxygenases introduce oxygen into the aromatic ring of 2,4-D, facilitating further breakdown. Dehalogenases remove halogen atoms from the molecule, while hydrolases and oxidases further degrade the compound. Together, these enzymes ensure the efficient conversion of 2,4-D into less harmful substances, benefiting the environment and organisms.^80^

#### 3.3.1.1 Laccases

Laccases (EC 1.10.3.2), multicopper oxidases, play a crucial role in the oxidation of various phenolic compounds, including toxic, carcinogenic, and mutagenic substances, as well as endocrine-disrupting chemicals often found in wastewater from industrial and conventional water-treatment processes.^70^ During the degradation process, laccase oxidizes the phenolic moieties of 2,4-D to form phenoxy radicals, which helps to detoxify the compound. In the context of 2,4-D degradation, laccases oxidize phenolic moieties to form phenoxy radicals. These radicals are highly reactive and can undergo further chemical transformations, including:^10,19–21^

- Radical coupling and polymerization
- Ring opening reactions
- Indirect C–Cl bond cleavage via destabilization of electron density near halogenated sites

The use of laccase-mediator systems (LMS) further extends the redox potential of the enzyme, enabling the oxidation of compounds that are not direct laccase substrates.^11^

A study by Serbent et al.^24^ investigated the treatment of two soils from Pennsylvania containing 2.8% (Soil 1) and 7.4% (Soil 2) organic matter, both polluted with 2,4-dichlorophenol (2,4-DCP). The study evaluated the effectiveness of laccase from *Trametes villosa*, free and immobilized on montmorillonite. In Soil 1, free and immobilized laccase could remove 100% of 2,4-DCP, regardless of moisture conditions. In Soil 2, immobilized laccase removed approximately 95% of 2,4-DCP across various moisture levels, while the free enzyme removed 55, 75, and 90% at 30, 55, and 100% of the soil’s maximum water retention capacity, respectively. It was noted that the enhanced activity of immobilized laccase came at the cost of a 23% reduction in enzyme activity during the immobilization process, which was roughly offset by a 30% increase in free laccase activity required to achieve similar remediation levels. Additionally, the use of immobilized laccase was more costly compared to free laccase from *T. villosa*. Laccase’s action in the degradation of chlorophenols has already been detected in studies involving fungal species such as *Pycnoporus cinnabarius*.^81^ *Trametes versicolor* and *Pleurotus ostreatus*.^82^

The degradation of 2,4-dichlorophenoxyacetic acid (2,4-D) and 2,4,5-trichlorophenoxyacetic acid (2,4,5-T) was investigated by three different fungi, focusing on the roles of laccases and P450-type cytochromes in this process.^28^ The white rot fungus *Ritidoporus* sp. FMD21, which exhibits high laccase activity, showed a positive correlation between laccase activity and the herbicide degradation rate. Specifically, when laccase activity doubled, the herbicide degradation rate also doubled. Additionally, two filamentous fungi isolated from soil contaminated with herbicides and dioxins at the Bien Hoa air base, identified as belonging to the genera Fusarium and *Verticillium* based on 18S rRNA gene sequences, demonstrated varying herbicide degradation rates. However, these fungi had deficient laccase activity, and no correlation was found between laccase activity and herbicide degradation rates. These findings suggest that white rot fungi likely use laccase and P450-type cytochromes for herbicide degradation, while the specific enzymes used by other fungi remain unclear.^81–83^

The dehalogenation is a potential step facilitated by laccase action, mediating some reactions within metabolic pathways.^84^ The action of this enzyme in specific pathways can lead to the production of less toxic compounds, such as catechol, anisole, and guaiacol, following dehalogenation. These products are helpful in various industrial processes and exhibit reduced toxicity compared to their chlorine-containing precursors.^85,86^

#### 3.3.1.2 Lignin Peroxidases and Manganese Peroxidases

Peroxidases, a class of heme-containing enzymes, are vital for the oxidation of various substrates, including phenolic compounds and aromatic pollutants like 2,4-D. They play a crucial role in the degradation of 2,4-D by generating reactive oxygen species (ROS), which facilitate the breakdown of chemical bonds and the mineralization of 2,4-D into less toxic metabolites.^87,88^

Fungal peroxidases, including manganese peroxidase (MnP), lignin peroxidase (LiP), and versatile peroxidase (VP), are heme-containing enzymes that use hydrogen peroxide (H_2_O_2_) as a cosubstrate to generate reactive intermediates.

- MnP catalyzes the oxidation of Mn^2+^ to Mn^3+^, which acts as a diffusible oxidizer for phenolic structures.
- LiP and VP generate high-valent iron-oxo species (e.g., Fe­(IV)=O), capable of oxidizing nonphenolic aromatic compounds and facilitating direct cleavage of C–Cl bonds.

These enzymes can break stable chemical bonds by producing reactive oxygen species (ROS) such as hydroxyl radicals and superoxide, which promote oxidative dechlorination.

The white-rot basidiomycete *Phanerochaete chrysosporium* was examined for its ability to mineralize 2,4-dichlorophenol (2,4-DCP) under secondary metabolic conditions. The study characterized fungal metabolites and oxidation products generated by purified lignin peroxidase and manganese peroxidase to elucidate the degradation pathway of 2,4-DCP.^89^

The degradation process involves a multistep oxidative dechlorination pathway leading to the formation of malonic acid. Initially, 2,4-DCP is oxidized to 2-chloro-1,4-benzoquinone by manganese peroxidase or lignin peroxidase. This intermediate is then reduced to 2-chloro-1,4-hydroquinone, which is further methylated to form 2-chloro-1,4-dimethoxybenzene, a substrate for lignin peroxidase.

Subsequent oxidation of 2-chloro-1,4-dimethoxybenzene by lignin peroxidase yields 2,5-dimethoxy-1,4-benzoquinone. This compound is reduced to 2,5-dimethoxy-1,4-hydroquinone, which is further oxidized by any peroxidase to produce 2,5-dihydroxy-1,4-benzoquinone. This product is then reduced to form the tetrahydroxy intermediate, 1,2,4,5-tetrahydroxybenzene. Peroxidase’s oxidative dechlorination throughout this pathway removes chlorine atoms from the substrate before ring cleavage occurs.

In addition to laccases and peroxidases, a comprehensive understanding of fungal 2,4-D biodegradation pathways requires considering several other enzyme families. Dioxygenases initiate aromatic-ring cleavage by incorporating molecular oxygen into chlorinated intermediates—this mechanism has been well-documented in fungal degradation studies.^9^ Dehalogenases, including fungal flavin-dependent monooxygenases, catalyze the removal of chlorine atoms, thus reducing toxicity and enabling further metabolism. Hydrolases break ester or ether bonds within recalcitrant metabolites, facilitating downstream degradation steps.^22^ Oxidoreductases such as cytochrome P450 monooxygenases (CYPs) are involved in oxidative dechlorination and hydroxylation; their function in 2,4-D degradation by fungi (e.g., *Umbelopsis isabellina*, *Phanerochaete chrysosporium*) has been confirmed via CYP inhibition assays.^23^ Finally, phenolic monooxygenases hydroxylate aromatic rings, often converting chlorinated phenols into catechol derivatives that are substrates for dioxygenases. Together, these enzyme systems—working synergistically with extracellular ligninolytic enzymes—paint a detailed picture of the complex metabolic networks fungi deploy in bioremediation of 2,4-D.

#### 3.3.1.3 Synergistic Action

A synergistic interaction between laccases and peroxidases plays a pivotal role in enhancing both the efficiency and scope of 2,4-D degradation. Laccases initiate the process by oxidizing phenolic moieties present in 2,4-D, generating highly reactive phenoxy radicals that destabilize the aromatic ring and create reactive intermediates susceptible to further transformation. Structurally, laccases possess multicopper centers that facilitate one-electron oxidation reactions, producing radicals capable of initiating the breakdown of otherwise recalcitrant compounds.^90^

Simultaneously, peroxidases—such as manganese peroxidase (MnP), lignin peroxidase (LiP), and versatile peroxidase (VP)—use hydrogen peroxide to generate reactive oxygen species (ROS) and high-valent iron-oxo intermediates. These powerful oxidants can directly attack the strong carbon–chlorine (C–Cl) bonds within the 2,4-D molecule, leading to oxidative dechlorination and ring cleavage. The structural features of peroxidases, including their heme prosthetic groups, enable these high-energy redox transformations.^37^ The combined action of laccase and peroxidases results in a synergistic effect, where the enzymatic activities of each enzyme complement and enhance the functions of the other. Mechanistically, the synergy arises because laccase-generated radicals increase the local redox potential and create reactive sites on the 2,4-D molecule, making C–Cl bonds more susceptible to attack by peroxidase-generated ROS. Meanwhile, peroxidases contribute to regenerating redox mediators and maintaining radical flux, sustaining laccase catalytic cycles. This cooperative mechanism accelerates degradation rates beyond what either enzyme achieves alone and broadens substrate specificity, enabling the breakdown of various chlorinated and aromatic pollutants.^27,64,91^

As a result, the laccase–peroxidase system effectively converts 2,4-D and its derivatives into simpler, less toxic metabolites such as chlorocatechols and quinones, which can undergo further metabolism or mineralization. This enzymatic synergy not only improves degradation efficiency but also enhances the applicability of fungal oxidative enzymes in bioremediation and environmental detoxification efforts, offering a versatile approach to tackling persistent organic pollutants.^37^

## 4 Applications of 3,4-D Byproducts

Several compounds can be derived from the degradation of 2,4-dichlorophenoxyacetic acid (2,4-D), including hydroquinones, benzoquinones, and dichloro-guaiacol. These metabolites, produced through microbial degradation, are gaining increasing attention for their potential value addition and utilization. Exploring these substances underscores a commitment to environmentally responsible waste management and opens innovative avenues for leveraging natural resources. This approach highlights the integration of environmental stewardship with technological innovation.^17,21^

Understanding the degradation pathways of 2,4-D by fungi is particularly crucial because while bacterial pathways for this herbicide are well-established, fungal pathways are less documented. Fungi, especially white rot fungi and various species within Ascomycota and Basidiomycota, exhibit significant potential for degrading 2,4-D. However, their specific mechanisms and pathways must be more thoroughly understood than their bacterial counterparts. Elucidating these fungal pathways is essential for several reasons.^15,56^

First, expanding the range of biological tools available for bioremediation is a significant advantage.^15^ While bacteria are effective, fungi offer several unique benefits.^12^ They can degrade a broader spectrum of pollutants and thrive in diverse environmental conditions. Fungi can penetrate deeper into soil matrices and organic matter, enhancing bioremediation efficiency in complex environments.^58,80,87^

Second, understanding fungal degradation pathways can lead to optimizing fungal strains for more effective bioremediation. Identifying the specific enzymes and intermediates involved in the degradation of 2,4-D makes it possible to enhance these processes through genetic engineering or by optimizing environmental conditions to favor the most effective degradation pathways.^92^

Additionally, elucidating these pathways allows for identifying and utilizing degradation intermediates in various applications. The intermediate compounds produced during the fungal degradation of 2,4-D could have potential uses in industrial and biotechnological processes. For example, confident intermediates might be precursors in synthesizing pharmaceuticals, agrochemicals, or other valuable compounds.^24^ Table 4 highlights some of the most recent studies on the applications of these intermediates.

**Table 4:** Reported 2,4-D Intermediates Applications

intermediate	application	ref(s)
hydroquinone	depigmenting agent in clinical for skin disorders	^93–95^
	antioxidant properties	^96^
	dye intermediate	^97^
	photographic reducer and developer	
	stabilizer in paints and varnishes	
	motor fuels and oils	
	inhibited biofilm formation by *Vibrio parahemolyticus*	^98^
catechol	adhesion for biomedical applications	^99^
	chelation of metals	^100^
	polystyrenic resin functionalized	
	antibacterial properties	^101^
	antimicrobial polymers	^102^
	hydrogel for skin wound healing	^103^
anisole	chemical, pharmaceutical, plastic, and pesticide industries	^104^
	pest control	^105^
	catalytic hydroprocessing	^106^
	cosmetic ingredient	^107^
	solvent	^108^
	fragrance materials and surfactants, preservatives in moisturizers and lipsticks	^109–111^
guaiacol	fungicidal	^112^
	deep eutectic solvent	^113^
	antimicrobial activity	^114^
	improve the aromatic profiles of wines	^115^
	preservative of cosmetic and sanitizing products	^116^
quinone	hydrogel to remove methyl blue	^117^
	antimalarial properties	^118^
	anticancer drugs	^119^
	DNA cleavage agents	^120^
	energy storage	^121^
guaiacol	fungicidal	^112^
	deep eutectic solvent	^113^
	antimicrobial activity	^114^
	improve the aromatic profiles of wines	^115^
	preservative of cosmetic and sanitizing products	^116^

The objective of in vitro degradation studies of 2,4-D often involves identifying secondary byproducts such as chlorohydroquinone, 3,5-dichlorocatechol, and 4,6-dichlororesorcinol. For instance, similar byproducts, including 2,4-dichlorophenol, 2- and 4-chlorophenol, 4-chlorocatechol, phenol, and catechol, in their studies on 2,4-D degradation,^48^ also observed the formation of byproducts like 2,4-dichlorophenol (2,4-DCP), 3,5-chlorocatechol, and chlorohydroquinone as intermediate steps in the breakdown process.

Studying these intermediates provides insight into the metabolic capabilities of fungi and their enzymatic systems and opens avenues for developing novel biocatalysts for both environmental and industrial applications. This knowledge can be harnessed to enhance bioremediation strategies and innovate new processes for utilizing fungal enzymes effectively.^47,85,95,122,123^

### 4.1 Quinones, Hydroquinone, and Benzoquinone

Quinones are crucial in many biological processes, primarily functioning as electron transport agents within redox cycles. These cycles are integral to various metabolic pathways, facilitating the transfer of electrons and driving essential biochemical reactions.^95,124^ This electron transfer capability is central to quinones’ roles in cellular respiration and photosynthesis, where they are vital participants in the electron transport chain, contributing to the production of ATP, the cell’s energy currency.^124–126^

Hydroquinones and benzoquinones, specific types of quinones, hold particular value in the pharmaceutical and food industries due to their potent antioxidant properties. As antioxidants, they neutralize free radicals—unstable molecules that can cause cellular damage—thereby combating oxidative stress. This function helps preserve the quality and extend the shelf life of various products. For example, in the food industry, antioxidants prevent the rancidity of oils and fats, maintaining food products’ nutritional and sensory qualities over time.^94,95^

In the pharmaceutical industry, hydroquinones and benzoquinones are utilized to develop medications that leverage their antioxidative properties to protect cells from oxidative damage. This oxidative stress is linked to numerous diseases, including cancer and neurodegenerative disorders. Therefore, these compounds are valuable in therapeutic applications to prevent or mitigate the effects of such conditions.^127^

Quinones, including ansamycin and geldanamycin, have demonstrated significant potential beyond their roles in electron transfer and antioxidant activity. Ansamycins, a class of antibiotics with benzoquinone structures, are known for their potent antimalarial properties. These compounds work by inhibiting heat shock protein 90 (Hsp90), crucial for the malaria parasite’s lifecycle. Geldamycin, a prominent member of this class, disrupts protein folding in the parasite, leading to its death. This makes ansamycins valuable in the ongoing fight against malaria, especially in regions where the disease remains endemic.^111^

Thymoquinone, another significant quinone, has garnered attention for its broad spectrum of biological activities, particularly its anti-inflammatory properties.^128^ Thymoquinone exerts its effects by modulating various signaling pathways and reducing the production of pro-inflammatory cytokines, making it a promising candidate for developing treatments for inflammatory diseases such as arthritis, asthma, and other chronic inflammatory conditions.

Beyond their biological activities, many quinones are utilized to enhance polymers, extending their utility to producing high-performance materials across various industries^124^ For instance, incorporating quinones into polymer matrices can significantly improve these materials’ thermal stability, mechanical strength, and resistance to degradation^121,126^ This makes them suitable for demanding aerospace, automotive, and electronics applications.^129^

Due to their versatile chemical structure, quinones are crucial in manufacturing dyes, pigments, and medicines. This structural adaptability allows for various modifications, making quinones invaluable for creating compounds with specific properties suited to multiple applications.^118,130^ In the dye and pigment industry, quinones are essential for producing vibrant and stable colors, which are vital for textiles, printing, and coatings. In medicine, quinones contribute to the synthesis of drugs by interacting with diverse biological targets, leveraging their redox properties to deliver therapeutic effects.^97,118,126^

One notable transformation is the conversion of quinones into chlorobenzoquinones, which are found in the degradation pathways of the herbicide 2,4-D (2,4-dichlorophenoxyacetic acid) by fungi.^39,89^ However, these chlorinated derivatives raise significant health concerns due to their high carcinogenic risk, highlighting the importance of monitoring and managing these compounds in environmental and industrial contexts.^131^

In Australia, the national introduction volume of hydroquinone ranges from 100 to 1000 tons per year. Hydroquinone is used in various applications, including photochemical reagents, photographic processing, resin and polymer manufacturing, and cosmetics. Although there is limited specific information about the introduction, use, and end use of p-benzoquinone and quinhydrone in Australia, these compounds are primarily used internationally as polymerization inhibitors for polyester resins and vinyl monomers. They also serve as intermediates in stabilizing adhesives, polymers, and resins, and as antioxidants in the rubber and food industries.^97^ Hydroquinone has been used as a reducing agent by photographic developers, though this use has significantly declined in recent years.^127^

As we explore the multifaceted potential of quinones, it becomes clear that their impact extends beyond environmental management and technological innovation to include transformative medical applications. This underscores the essential role of quinones in advancing scientific knowledge and addressing global challenges.^93,95,126^

### 4.2 Chlorophenols

Chlorophenols are a diverse group of chemicals formed through the electrophilic halogenation of phenol with chlorine, resulting in five basic types and 19 distinct chlorophenols. These compounds have various applications, including pesticides, herbicides, antiseptics, and disinfectants.^131^ Among them, pentachlorophenol is particularly significant due to its extensive use as a fungicide. First registered in the United States in 1936 as a wood preservative, pentachlorophenol has since been incorporated into products such as ropes, paints, adhesives, canvas, insulation, and brick walls.^132^

Another prominent chlorophenol is 2,4-dichlorophenol (2,4-DCP), a primary degradation product of the herbicide 2,4-D. This compound produces pesticides, herbicides, and antiseptics. The widespread application of chlorophenols in industrial and agricultural settings underscores their versatility.

However, the chlorophenols’ extensive use and persistence in the environment pose significant health and environmental risks. The United States Environmental Protection Agency (EPA) lists chlorinated phenols as a priority pollutant due to their high toxicity and carcinogenicity.^131,133^ This designation highlights the need for stringent regulation and monitoring to mitigate their harmful effects.

From an environmental perspective, the aerobic degradation of chlorophenols can produce less harmful compounds such as catechols and guaiacols.^114,123^ These degradation products are particularly relevant in the cellulose industry, where they participate in various processes.^84,134^ Effective degradation of chlorophenols is essential for reducing their environmental footprint and managing their presence in industrial waste.^133^

In summary, while chlorophenols have valuable applications across multiple sectors, their potential health risks and environmental impact necessitate ongoing research and regulatory measures to ensure their safe use and disposal.^132,133^ Balancing their utility with ecological protection remains a critical focus for scientists and policymakers.

### 4.3 Catechols

Catechols have emerged as valuable compounds in various industrial applications due to their adhesive properties and alignment with green chemistry principles. In the textile industry, catechols offer a sustainable alternative to conventional dyeing methods, addressing environmental challenges associated with traditional processes. By incorporating catechols into textile coloration, it is possible to achieve more environmentally friendly production techniques, thereby reducing the ecological impact of dyeing.^99,122,135^

The versatility of catechols stems from their capacity to participate in various reversible interactions, including hydrogen bonding, π–π electron interactions, cation-π-π interactions, coordination with metal oxide surfaces and metal ions, and covalent bond formation. When integrated into polymers, catechols impart unique chemical reactivity, making them suitable for advanced material design. This includes applications in adhesives, antifouling coatings, drug carriers, and antimicrobial polymers.^136–138^ Their ability to adhere firmly to various surfaces enhances their practicality in diverse applications.^100,102,103^

In addition to their adhesive capabilities, plant-derived compounds such as tannic acid (TA) and catechin exhibit similar intermolecular interactions and cross-linking properties as catechol.^135^ These compounds are often utilized as surface anchoring agents to improve interfacial bonding. Recent studies have also underscored the role of catechols in generating reactive oxygen species (ROS) during their oxidation process. These ROS are effective broad-spectrum biocides, presenting valuable opportunities in industrial and biomedical fields.^85,139,140^

Furthermore, chemical modifications of catechols, including halogenation and the incorporation of polyphenols such as TA, curcumin, catechin, and procyanidin, enhance their intrinsic antimicrobial properties. These modifications boost the biocidal efficacy of catechols and broaden their application in developing antimicrobial coatings and materials that can inhibit the growth of harmful microorganisms.^101,135,141^

### 4.4 Anisole

Anisole is recognized for its use in producing dyes, cosmetics, and fragrances due to its pleasant aroma and reactivity.^142,143^ Its versatility extends beyond these applications, serving as a reactive intermediate in various industrial sectors.^108^

In the agrochemical and petrochemical industries, anisole is used as an additive to gasoline to enhance octane efficiency and fuel performance^105,108,110,144^ By improving the combustion properties of gasoline, anisole contributes to more efficient engine operation and reduced knocking.^145–147^ This application underscores its significance in enhancing fuel quality and efficiency.^148^

Additionally, anisole functions as an antioxidant in the production of greases and oils, which helps prevent oxidation and extends these products’ shelf life and performance. In plastics and polymers, anisole acts as a stabilizer, maintaining the integrity and durability of materials during processing and use.^147^ These roles demonstrate anisole’s critical contribution to improving the properties and longevity of various industrial products.^129,149^

Recent research has also highlighted the innovative use of anisole and its derivatives, such as 2-bromoanisole and 3-bromoanisole, as novel additives for overcharge protection in lithium-ion batteries.^150^ These compounds are being studied for their potential to enhance battery safety and performance by preventing overcharging, which can lead to overheating and failure.^144^ This emerging application illustrates the ongoing exploration of anisole’s potential in advanced technological fields, particularly energy storage.^149^

## 5 Conclusions

Utilizing enzymes such as laccase and peroxidases in the degradation of 2,4-dichlorophenoxyacetic acid (2,4-D) represents a sustainable and effective strategy for environmental remediation. The synergistic action of these enzymes significantly enhances the breakdown of 2,4-D, facilitating the detoxification of pollutants and contributing to the production of valuable industrial byproducts. Enzymes like laccase and peroxidases are crucial in degrading 2,4-D, offering an environmentally friendly alternative to traditional chemical and physical remediation methods. Their combined action promotes efficient pollutant removal and contributes to reducing environmental contamination.

The degradation of 2,4-D by filamentous fungi generates various byproducts with considerable industrial and commercial potential. These byproducts, which include hydroquinones, benzoquinones, and other metabolites, present opportunities for use in pharmaceuticals, cosmetics, and chemicals, thereby supporting a circular economy. Understanding and harnessing these complex biochemical processes aids environmental preservation and drives technological innovation. The transformation of waste products into valuable resources exemplifies biotechnology’s potential to address global ecological and economic challenges.

These findings underscore the ability of biotechnology to bridge the gap between environmental sustainability and industrial advancement. By converting pollutants into valuable materials, we can achieve a balanced development vision that meets human and ecological needs. This harmonious approach highlights the promise of integrating sustainable practices with technological progress to foster a more resilient and resource-efficient future.

### 5.1 Future Perspectives

Exploring fungal degradation pathways for 2,4-dichlorophenoxyacetic acid (2,4-D) is promising for advancing bioremediation technologies. To fully capitalize on this potential, future research should focus on several key areas:

a. *Elucidation of Metabolic Pathways:* Detailed studies are needed to unravel the specific metabolic pathways employed by fungi in degrading 2,4-D. They understand key enzymes’ roles, such as laccases and peroxidases. These enzymes are pivotal in initiating and facilitating the breakdown 2,4-D into less harmful substances. A comprehensive knowledge of these mechanisms will enhance our ability to harness and optimize fungal capabilities for environmental remediation.
b. *Characterization of Intermediate Compounds:* Identifying and characterizing the intermediate compounds produced during the fungal degradation of 2,4-D is essential. These intermediates could have valuable applications in various industries, including pharmaceuticals, agrochemicals, and other sectors. Developing efficient methods to isolate and utilize these intermediates could add economic value to bioremediation processes and create new opportunities for resource recovery.
c. *Integration with Sustainable Technologies:* Combining fungal bioremediation with other sustainable technologies, such as phytoremediation and microbial consortia, could enhance pollutant degradation and promote ecosystem recovery. For example, integrating fungi with plants that can uptake and detoxify pollutants or with microbial communities that can break down a broader range of contaminants could lead to more comprehensive and effective remediation strategies.
d. *Collaborative Research Efforts:* Collaborative efforts among microbiologists, environmental scientists, and biotechnologists will be crucial in developing and implementing these strategies. Such interdisciplinary approaches can drive innovation and lead to comprehensive solutions that address complex environmental challenges.

By focusing on these areas, future research can advance our understanding of fungal bioremediation processes, optimize their application in environmental remediation, and explore the potential of fungal metabolites in industrial applications.
